# Phosphoinositides and intracellular calcium signaling: novel insights into phosphoinositides and calcium coupling as negative regulators of cellular signaling

**DOI:** 10.1038/s12276-023-01067-0

**Published:** 2023-08-01

**Authors:** Byung-Chul Oh

**Affiliations:** Department of Physiology, Lee Gil Ya Cancer and Diabetes Institute, Gachon College of Medicine, Incheon, 21999 Republic of Korea

**Keywords:** Calcium signalling, Obesity, Mechanisms of disease

## Abstract

Intracellular calcium (Ca^2+^) and phosphoinositides (PIPs) are crucial for regulating cellular activities such as metabolism and cell survival. Cells maintain precise intracellular Ca^2+^ and PIP levels via the actions of a complex system of Ca^2+^ channels, transporters, Ca^2+^ ATPases, and signaling effectors, including specific lipid kinases, phosphatases, and phospholipases. Recent research has shed light on the complex interplay between Ca^2+^ and PIP signaling, suggesting that elevated intracellular Ca^2+^ levels negatively regulate PIP signaling by inhibiting the membrane localization of PIP-binding proteins carrying specific domains, such as the pleckstrin homology (PH) and Ca^2+^-independent C2 domains. This dysregulation is often associated with cancer and metabolic diseases. PIPs recruit various proteins with PH domains to the plasma membrane in response to growth hormones, which activate signaling pathways regulating metabolism, cell survival, and growth. However, abnormal PIP signaling in cancer cells triggers consistent membrane localization and activation of PIP-binding proteins. In the context of obesity, an excessive intracellular Ca^2+^ level prevents the membrane localization of the PIP-binding proteins AKT, IRS1, and PLCδ via Ca^2+^-PIPs, contributing to insulin resistance and other metabolic diseases. Furthermore, an excessive intracellular Ca^2+^ level can cause functional defects in subcellular organelles such as the endoplasmic reticulum (ER), lysosomes, and mitochondria, causing metabolic diseases. This review explores how intracellular Ca^2+^ overload negatively regulates the membrane localization of PIP-binding proteins.

## Metabolic disease: interactions between calcium and lipid-binding proteins

The intersection between two major signaling mechanisms involving calcium and phospholipids may contribute to chronic medical conditions, including diabetes and cardiovascular disease. Many cellular functions are coordinated by a family of phospholipids known as phosphoinositides (PIP), which selectively recruit certain signaling proteins to the cell membrane for activation. This process is directly involved in modulating metabolic activity. Byung-Chul Oh, Gachon College of Medicine, Incheon, South Korea, reviews how PIP-based signaling is influenced by disturbances in a second pathway regulating calcium ion flux. After reviewing the two signaling pathways, the author notes that abnormally elevated calcium ion levels in cells generate a stress response that can contribute to obesity and diabetes. Oh highlights evidence that this increased calcium creates conditions that actively impede the recruitment of PIP-binding proteins to the cell membrane, further fueling cardiovascular and metabolic dysfunction.

## Introduction

Phosphoinositides (PIPs) are critical signaling lipids in the cytoplasmic leaflet of eukaryotic membranes^1,2^. PIPs are widely recognized as essential regulators of many biological and cellular processes that broadly impact nearly all aspects of membrane function and dynamics. Reversible phosphorylation and dephosphorylation of the PIP inositol ring by PIP kinases and phosphatases, respectively, generate seven biologically distinct phosphoinositide species^3,4^: PI(3)P, PI(4)P, PI(5)P, PI(3,4)P_2_, PI(4,5)P_2_, PI(3,5)P_2_, and PI(3,4,5)P_3_. PIP kinases catalyze phosphorylation at positions 3, 4, or 5 of the inositol ring of PIP, whereas lipid phosphatases such as phosphatase and tensin homolog on chromosome 10 (PTEN) and SH2-containing inositol 5′-phosphatase (SHIP) catalyze dephosphorylation at positions 3 or 5 of the PIP inositol ring^5^. Thus, lipid kinases and phosphatases precisely regulate the turnover of these seven interconvertible PIPs at dedicated membrane compartments in response to insulin, growth factors, and cytokines^2,4,6–8^. These seven PIPs are lipids in which proteins dock, which is mediated through specific PIP-binding domains^1,2,8,9^, including the pleckstrin homology (PH); C2; AP180 N-terminal homology (ANTH); phox homology (PX); Bin, amphiphysin, and Rvs (BAR); Fab1, yoTb, vac1, and eeA1 (FYEV); β-propeller that binds phosphoinositides (PROPPIN); and epsin N-terminal homology (ENTH) domains. Recognition of signaling proteins with PIP-binding domains by specific PIPs is essential for many cellular processes, including cell proliferation, vesicle trafficking, and homeostasis maintenance.

Among PIP-binding domains, PH domains are effectors that recognize the lipid second messengers PI(3,4)P_2_ or PI(3,4,5)P_3_, which are transiently generated by phosphatidylinositol (PI) 3-kinase (PI3K)^1,10^ in response to insulin, growth factors, and cytokines. In addition, insulin- or growth factor-stimulated PI3K products are critical for signal-dependent membrane recruitment of the PH domain-carrying several kinases, such as protein kinase B (AKT), Bruton’s tyrosine kinase (BTK)^11,12^, interleukin-2-inducible T-cell kinase (ITK)^13^, and general receptor for phosphoinositide 1 (GRP1)^14^, in response to hormone and cytokine stimulation. Mutations in the PH domain that disrupt PI(3,4)P_2_ or PI(3,4,5)P_3_ binding have been reported to cause severe signaling defects such as those causing X-linked agammaglobulinemia in humans and X-linked immunodeficiency in mice^11,15^. In contrast, PH domain mutations that promote constitutive plasma membrane localization of AKT can cause cancer^16^. These studies suggest that membrane localization of PH domains through the specific recognition of PI(3,4,5)P_3_ is crucial for signal transduction. Moreover, dephosphorylation of PI(3,4,5)P_3_ via phosphoinositide phosphatases^6,7^, such as PTEN and SHIP2, transiently attenuates PI(3,4,5)P_3_ binding specificity and affinity for PH domains. The orchestrated binding of PH domains to PIPs is critical for insulin-stimulated membrane targeting and signal transduction.

Intracellular Ca^2+^ is a universal and versatile signaling molecule that controls many cellular processes, including muscle contraction, neurotransmission, hormone secretion, organelle communication, cell metabolism, and cell growth^17^. Given the versatility and diversity of Ca^2+^ functions, intracellular Ca^2+^ homeostasis is tightly regulated by spatiotemporally coordinated interactions of plasma membrane channels and transporters, as well as ER and mitochondrial Ca^2+^ transporters and pumps^17–21^. Thus, dysregulation of Ca^2+^ channels, pumps, and transporters in the plasma membrane, ER, and mitochondria has been shown to play an essential role in insulin resistance in diabetes and obesity^19,20,22–24^. Furthermore, obesity-induced ER dysfunction leads to Ca^2+^ depletion from ER stores and activation of ER stress and the unfolded protein response (UPR)^19,20,24–26^. Chronic Ca^2+^ depletion in the ER results in intracellular and mitochondrial Ca^2+^ overload^27^, which may further exacerbate mitochondrial dysfunction, such as a reduction in mitochondrial adenosine triphosphate (ATP) production and increased mitochondrial reactive oxygen species (ROS) production^28^.

Ca^2+^ and PIPs are versatile signaling molecules associated with many regulatory cellular pathways. Although the mechanistic details of Ca^2+^ and PIP coupling are only now beginning to be understood^29,30^, it is evident that through crosstalk, synergy, and negative feedback mechanisms, intracellular Ca^2+^ fine-tunes PIP signaling to mediate complex metabolic responses. Although alterations in intracellular Ca^2+^ homeostasis have been shown to be key pathophysiological events in insulin resistance, obesity, and type 2 diabetes, we discuss the molecular mechanism underlying intracellular Ca^2+^-mediated inhibition of PH-domain-carrying protein targeting the plasma membrane. We will also discuss the potential role of intracellular Ca^2+^ as a contributing factor to the inhibition of PIP signaling, especially signaling mediated through the targeting of C2 and PH domain–carrying proteins to the membrane. Throughout this review, we highlight the expression and function of various Ca^2+^ channels, pumps, and transporters in the plasma membrane, ER, and mitochondria and describe their roles in the pathogenesis of metabolic diseases.

## Biosynthesis and metabolism of PIPs

**Fig. 1:**
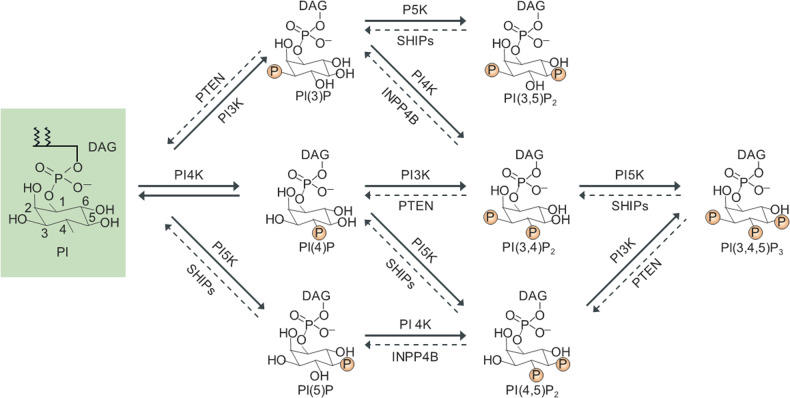
Structures of phosphoinositol and seven interconvertible phosphoinositides found in mammalian cells. Specific lipid kinases and phosphatases are phosphorylated or dephosphorylated at hydroxyl group positions 3, 4, and 5 within the inositol ring of phosphoinositol. PI phosphatidyl inositol, DAG diacylglycerol, PTEN phosphatase and tensin homolog on chromosome 10, SHIP SH2-containing inositol 5′-phosphatase, PI3K phosphatidylinositol 3-kinase, PI4K phosphatidylinositol 4-kinase, PI5K phosphatidylinositol 5-kinase, INPP4B inositol polyphosphate-4-phosphatase, type II.

The primary acidic phospholipids in mammalian cell membranes are phosphatidylserine, phosphatidic acid, and phosphatidylinositol (PI) phosphate. PIPs constitute only a small fraction of cellular phospholipids derived from PI but control most aspects of cell physiology^2,9^. This versatility of PIPs is a result of the chemistry of the *myo*-inositol moiety, which is attached to diacylglycerol (DAG) via a diester phosphate at the D-1 position, with five free hydroxyls occupying the other ring positions. Hydroxyl groups in the inositol ring at positions 3, 4, and 5 are phosphorylated or dephosphorylated by lipid kinases and phosphatases, respectively, generating seven distinct PIP species, PI(3)P, PI(4)P, PI(5)P, PI(3,4)P_2_, PI(3,5)P_2_, PI(4,5)P_2_, and PI(3,4,5)_3_ (Fig. 1). Specific lipid kinases and phosphatases regulate the turnover of these seven interconvertible PIPs at dedicated membrane compartments in response to stimuli^2^. Each of the seven phosphoinositides has a unique subcellular distribution with a predominant localization in subsets of membranes^2^. Despite their low abundance, the distinctive intracellular distribution and high turnover of PIPs make these lipids key mediators of signaling events in all cellular compartments, where they function as spatiotemporal cues to modulate membrane protein recruitment. Thus, PIPs play essential roles in directing the membrane localization of phospholipid-binding domains in proteins involved in signal transduction, cytoskeleton reorganization, membrane dynamics, and vesicular trafficking. Generally, negatively charged inositol phosphate groups of membrane PIPs interact electrostatically with positively charged PIP-binding domains with high affinity; these domains include annexin, BAR, C2, Fer/Cdc42-interacting Protein-4 (CIP4), homology-Bin-Amphiphysin-Rvs (F-BAR), four-point one, ezrin, radixin, and moesin (FERM), FYVE, PH, PX, ENTH/ANTH, Tubby, and PROPPINs^1,31^. These domains function as docking sites through which signaling proteins are targeted for transport to specific subcellular locations and might directly regulate protein function. In addition to PH domains, many other domains, including ANTH, PX, BAR, FYEV, PROPPIN, and ENTH domains, interact with various PIPs and are reviewed elsewhere^1,2^.

## PIPs recruit PH domain–carrying proteins to the plasma membrane and regulate diverse cellular responses

**Fig. 2:**
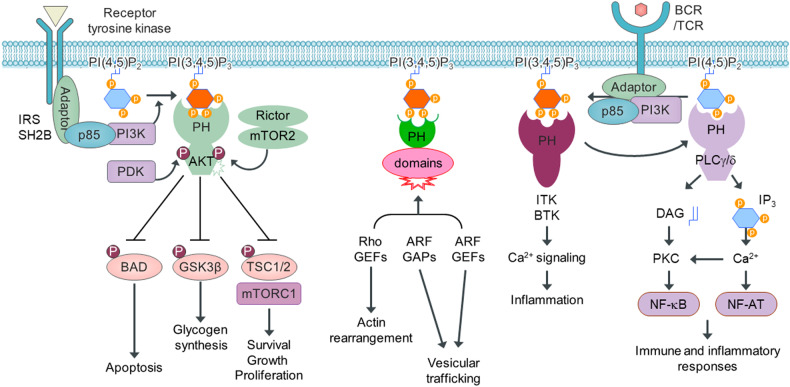
PIPs recruit PH domain–carrying proteins to the plasma membrane and regulate diverse cellular functions. Upon activation, PI3K phosphorylates PI(4,5)P_2_ to generate PI(3,4,5)P_3_. Activation of PI3K by either a receptor tyrosine kinase, B-cell receptor (BCR)/T-cell receptor (TCR), or cytokine receptor enables it to phosphorylate PI(4,5)P_2_, resulting in PI(3,4,5)P_3_, which recruits PH domain–carrying signaling proteins, such as PDK, AKT, Rho guanine nucleotide exchange factors (GEFs), ADP-ribosylation factor (ARF) GAPs, ARF GEFs, ITK, and BTK, to the plasma membrane. These PH domain–carrying proteins are then activated at the plasma membrane and regulate various cellular functions, including cell survival, proliferation, cytoskeleton rearrangement, intracellular vesicle trafficking, cell metabolism, and immune and inflammatory responses.

PH domains are small protein modules found in major regulators of vesicle biogenesis and are thus involved in intracellular trafficking; the proteins carrying these domains include pleckstrin, the AKT/RAC family serine/threonine kinases, Btk/Itk/Tec subfamily nonreceptor tyrosine kinases, NrKA/NrKB protein kinases, the breakpoint cluster region protein (BCR), the Rho/Rac guanine nucleotide exchange factor FGDs, insulin receptor substrates (IRSs), PLCs, the Rho family of guanosine triphosphatases (GTPases), the Ras GTPase-activating protein (RasGAP), oxysterol-binding proteins, ceramide kinase, G protein receptor kinases, Src homology 2B (SH2B) adapter proteins involved in tyrosine receptor kinase family member binding, KIF1A, and ADP-ribosylation factors (ARFs)^12,13,15,32–36^. The PH domain comprises approximately 120 amino acids and can bind PIPs, which mediate intracellular signaling, trafficking, and regulation^37^. The crystal structures of several PH domains have revealed how they recognize the phosphate groups in PIPs and anchor them through electrostatic interactions via positively charged side chains^38^. Based on in vitro and in vivo assays, PH domains can be classified as showing specific and high affinity or broad and low affinity for PIPs^39^. PH domains are important effectors for the phospholipid second messengers PI(3,4)P_2_ and PI(3,4,5)P_3_. These second messengers are transiently generated by cell surface receptor-stimulated PI3K activation following insulin, growth factor, or cytokine stimulation^3^. Therefore, the PH domains of kinases downstream of PI3K, such as AKT^32^, BTK^11,12^, ITK^13^, and GRP1^14^, recognize either PI(3,4)P_2_, PI(3,4,5)P_3_ or both with remarkable specificity and affinity. This precise recognition of the PI3K products PI(3,4)P_2_ and PI(3,4,5)P_3_ by PH domains is crucial for signaling-dependent membrane recruitment of kinases such as AKT, BTK, ITK, and GRP1 in response to hormone and cytokine stimulation^40^. In addition, the PH domain of PLCδ specifically recognizes membrane PI(4,5)P_2_^35^ (Fig. 2). Interestingly, mutations in the PH domain of BTK, which abolish PI(3,4,5)P_3_ binding, cause profound signaling defects, manifesting as, for example, X-linked agammaglobulinemia in humans and X-linked immunodeficiency in mice^41^. In contrast, the PH domain E17K mutation in AKT^16^ and the PH domain E41K mutation in BTK^15^ led to constitutive membrane recruitment and hyperactivation of AKT and BTK, respectively, in the cancer context. However, other PH domains, such as IRSs, Sex7, SH2B, and CDC24P, exhibit broad binding specificity for all seven PIPs^33^, suggesting that PH domains without clear specificity or affinity for PI(3)P, PI(4)P, PI(5)P, or PI(3,5)P_2_ play essential roles in the membrane localization of PH domain-carrying proteins^34^.

## The PI3K-AKT pathway and major upstream effectors in the insulin signaling network

**Fig. 3:**
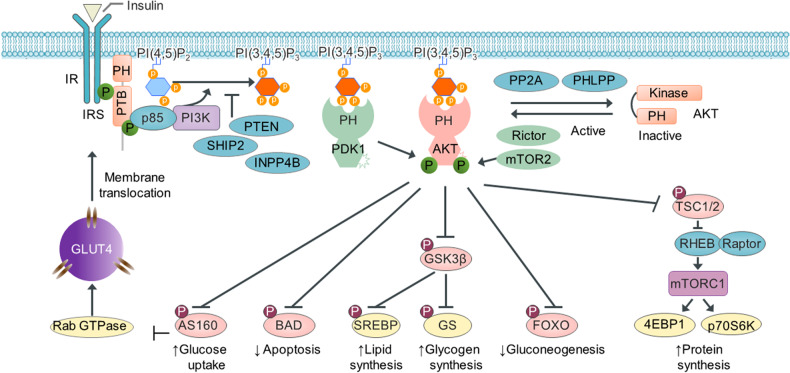
The PI3K-AKT pathway and major upstream effectors in the signaling network. In the presence of insulin, the insulin receptor (IR) autophosphorylates its cytosolic tyrosine kinase, which then phosphorylates IRS proteins and creates binding sites that recruit p85 and the catalytic subunit of PI3K to the plasma membrane. PI3K phosphorylates PI(4,5)P_2_ to produce PI(3,4,5)P_3_, which recruits PDK1 and AKT to the plasma membrane, and at the plasma membrane, AKT is phosphorylated at T308 and S473 by PDK1/2 and mammalian target of rapamycin complex 2 (mTORC2), respectively. AKT signaling promotes glucose uptake and lipid, glycogen, and protein synthesis via the phosphorylation of the GTPase-activating AS160, GSK3β, and tuberous sclerosis complex (TSC1/2) complex. In contrast, AKT signaling inhibits apoptosis and gluconeogenesis via the phosphorylation of proapoptotic Bcl-2 protein (BAD) and forkhead box O family transcription factor (FOXOs). Alternatively, negative regulators of PI3K/AKT include PTEN, SHIP, inositol polyphosphate-4-phosphatase (INPP4B), phosphatase 2A (PP2A), and PH-domain leucine-rich-repeat protein phosphatase (PHLPP). The lipid phosphatase PTEN dephosphorylates the 3-phosphate of PI(3,4)P_2_ and PI(3,4,5)P_3_ to generate PI(4)P and PI(4,5)P_2._ SHIP2 dephosphorylates the 5-phosphate of PI(3,4,5)P_3_ to yield PI(3,4)P_2_, and INPP4B dephosphorylates the 4-phosphate of PI(3,4)P_2_ and PI(3,4,5)P_3_ to generate PI(3)P and PI(3,5)P_2_. Ultimately, a lipid phosphatase decreases the PI(3,4,5)P3 level and inhibits the membrane localization of PDK1 and AKT, inhibiting insulin signaling. Moreover, the protein phosphatases PP2A and PHLPP dephosphorylate and inhibit AKT activity. GLUT4, glucose transporter-4; RHEB, the small GTPase Ras homolog enriched in the brain; Raptor, regulatory associated protein of mTOR; Rictor, the rapamycin-insensitive companion of mTOR; 4EBthe P1, eukaryotic translation initiation factor 4E-binding protein 1; P70S6k, p70 ribosomal protein S6 kinase.

Insulin is a major anabolic hormone that controls critical energy metabolism processes, such as glucose and lipid metabolism. Insulin stimulates cell growth and differentiation and regulates the metabolism of carbohydrates, fats, and proteins in adipose tissue, the liver, and muscles by stimulating lipogenesis, glycogen synthesis, and protein synthesis and inhibiting autophagy, gluconeogenesis, glycogenolysis, protein degradation, and lipolysis^42^ (Fig. 3). The beta cells in pancreatic islets sense blood sugar levels and secrete insulin into the blood in response to food intake and high blood glucose levels. Insulin binds to its receptor, which results in receptor autophosphorylation at tyrosine residues. These tyrosine residues are docking sites for the phosphotyrosine-binding (PTB) domain of IRS proteins (IRS-1, IRS-2, and IRS-3), and then, tyrosine phosphorylation is mediated via an insulin receptor tyrosine kinase. Tyrosine-phosphorylated IRS is a docking site for the SH2 domain of the p85 regulatory subunit of PI3K and various signaling partners, such as Grab2 and Fyn.

The catalytic subunit of PI3K, p110, then catalyzes the phosphorylation of the hydroxyl group at position 3 in PI(4,5)P_2_ to generate PI(3,4,5)P_3_^43^, which recruits cytosolic and inactive AKT, a key downstream effector, to the plasma membrane through the AKT PH domain^3,4^. This action leads to the phosphorylation and activation of AKT by phosphoinositide-dependent protein kinase 1 (PDK1) and mammalian target of rapamycin (mTOR) complex 2 (mTORC2)^44^. Activated AKT functions as a key convergence point in the PI(3,4,5)P_3_-mediated insulin signaling pathway by phosphorylating the GTPase-activating protein AKT substrate of 160 kDa (AS160), cyclic AMP (cAMP) response element-binding protein (CREB), the enzyme glycogen synthase kinase 3β (GSK3β), forkhead transcription factors (FOXOs), sterol regulatory element-binding protein (SREBP), and tuberous sclerosis complex 2 (TSC2)^42^. Once activated, AKT phosphorylates and inactivates GSK3β, which leads to the activation of glycogen synthase, a major substrate of GSK3, resulting in glycogen synthesis^45^. Active AKT stimulates glucose uptake in the liver via the phosphorylation of the GTPase-activating protein AS160, which is critical for the translocation of glucose transporter 4 (GLUT4)-carrying vesicles to the plasma membrane^46^. AKT also regulates cell survival by inhibiting several proapoptotic factors, including proapoptotic Bcl-2 protein (BAD)^47^. Moreover, AKT phosphorylates and directly inhibits FOXO transcription factors^48^, which are sequestered in the cytoplasm via their interactions with 14-3-3 proteins to inhibit gluconeogenesis and autophagy in the liver. Insulin signaling induces fatty acid and cholesterol synthesis by regulating SREBP transcription factor activity^49^. AKT effectively activates the mTOR pathway through its phosphorylation of TSC1/2, a negative regulator of mTOR. Thus, AKT-activated mTOR stimulates protein synthesis by phosphorylating p70 ribosomal protein S6 kinase (p70S6K) and eukaryotic translation initiation factor 4E binding protein 1 (4EBP1)^50^ (Fig. 3). Importantly, PIPs play key roles in the PI3K/AKT insulin signaling pathway and glucose and lipid metabolism; dysregulation of these signaling pathways is associated with various metabolic diseases, including diabetes, obesity, inflammatory conditions, and cardiovascular disease.

## Negative regulators of PI3K/AKT signaling

Activation of PI3K at the plasma membrane stimulates the phosphorylation of the phospholipid substrate PI(4)P or PI(4,5)P_2_ to yield PI(3,4)P_2_ and PI(3,4,5)P_3_. The cellular concentrations of PI(3,4)P_2_ and PI(3,4,5)P_3_ are modulated by two types of inositol-specific phosphatases in the cell: PTEN and SHIP1 and SHIP2^5,7^. PI3K signaling is attenuated by PTEN, which dephosphorylates the 3-phosphate in PI(3,4)P_2_ and in PI(3,4,5)P_3_ to generate PI(4)P and PI(4,5)P_2_, respectively. Ultimately, PTEN decreases plasma membrane PI(3,4)P_2_ and PI(3,4,5)P_3_ levels, which drives insulin signaling. PTEN eliminates docking sites for recruited downstream PH domain-carrying effector proteins that engage with the PI3K products PI(3,4)P_2_ and PI(3,4,5)P_3_. Thus, PTEN suppresses PI3K-stimulated ATK, BTK, ITK, and GRP1 by disrupting the membrane localization of effector molecules. PTEN-deficient mice have been shown to exhibit increased insulin sensitivity and glucose tolerance in peripheral tissues^51^. Moreover, the knockdown of PTEN using antisense oligonucleotides reversed hyperglycemia in diabetic ob/ob mice^52^. In contrast, SHIP1 and SHIP2 dephosphorylate the 5-phosphate of PI(3,4,5)P_3_ to yield PI(3,4)P_2_. SHIP1 expression is exclusive to hematopoietic cells and thus plays a key role in immune system regulation. SHIP2 is widely expressed in most cells and negatively regulates insulin signaling by inhibiting the membrane localization of AKT by decreasing the PI(3,4,5)P_3_ level. Mice lacking SHIP2 show increased glucose tolerance and reduced insulin sensitivity after increased glucose uptake and glycogen synthesis^6^, suggesting that SHIP2 is a critical negative regulator of insulin signaling and sensitivity (Fig. 3). Together, these results suggest that PI(3,4,5)P_3_-mediated membrane targeting of effector molecules plays an important role in insulin signaling.

In addition to lipid-specific phosphatases, PI3K/AKT signaling is negatively regulated by O-linked-β-N-acetylglucosamine transferase [O-GlcNAc (OGT)]^53^ and the mitochondrial protein prohibitin (PHB)^54^. Following insulin treatment, OGT was translocated to the plasma membrane mediated by PI(3,4,5)P_3_, inhibiting the phosphorylation of AKT. OGT and PHB physically interact^55^ and are phosphorylated by the insulin receptor^56^ and AKT^57^, respectively. OGT and PHB are thought to be components of protein complexes that negatively regulate insulin signaling. Moreover, AKT activity is regulated by several protein phosphatases, such as protein phosphatase-2A (PP2A) and PH-domain leucine-rich repeat protein phosphatase (PHLPP), which directly dephosphorylate and inhibit AKT activation^58,59^ (Fig. 3).

## Regulation of Ca^2+^ homeostasis

**Fig. 4:**
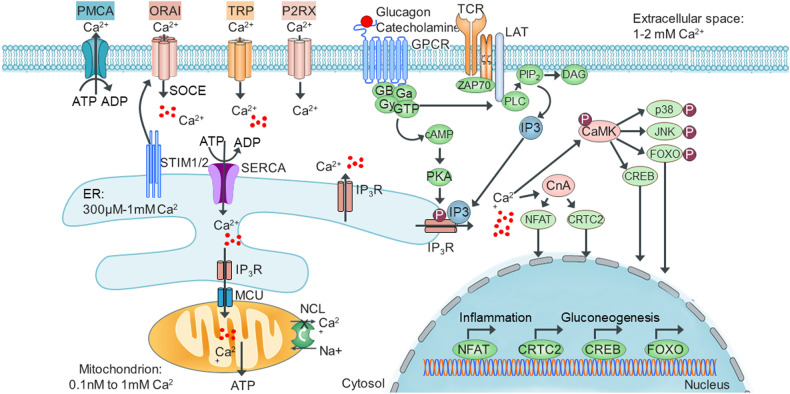
Regulation of Ca^2+^ homeostasis. Cytoplasmic, ER, and mitochondrial Ca^2+^ homeostasis is maintained by the actions of transporters and pumps, including PMCAs, SERCAs, and NCLX. Other plasma membrane channels, such as TRP channels and P2RXs, also mediate Ca^2+^ signaling during GPRC activation. Stimulation of G protein-coupled receptors (GPCRs) by glucagon and catecholamines or the TCR by specific antigens leads to the activation of PLC, resulting in I(1,4,5)P_3_ (IP3) and DAG production. IP3 binds to IP3Rs, stimulating Ca^2+^ release from ER Ca^2+^ stores. A decrease in ER Ca^2+^ level is sensed by the low-affinity EF-hands of STIM1/2, which activates ORAI1 proteins at the plasma membrane, inducing SOCE. IP3R mediates the release of Ca^2+^, which is transferred into mitochondria through the MCU at highly specialized membrane contact sites, mitochondrion-associated membranes that effectively enhance bioenergetics and ATP production. Alternatively, GPCR activation leads to cAMP production, activating PKA, which phosphorylates IP3R and thus activates its activity. In the cytosol, increased intracellular Ca^2+^ levels activate two main Ca^2+^ sensors, Ca^2+^/calmodulin-dependent protein kinase (CaMK) and calcineurin (CnA). CaMKs directly phosphorylate many targets, including the inflammatory proteins JNK and p38 and transcription factors such as FOXO and cAMP response element-binding protein (CREB). High intracellular Ca^2+^ levels lead to the activation of CaN, which regulates the activity of at least two crucial transcription pathways involving the nuclear factor of activated T cells (NFAT) and CREB-regulated transcription coactivator 2 (CRTC2). TRP nonselective transient receptor potential, P2RX purinergic ionotropic receptor, MCU mitochondrial Ca^2+^ uniporter, PMCA plasma membrane Ca^2+^ ATPase, SERCA sarcoplasmic/ER Ca2+ ATPase, NCLX mitochondrial Na^+^/Ca^2+^/Li^+^ exchanger.

Ca^2+^ is an abundant divalent ion in the body and is primarily stored in bones. As an electrolyte, Ca^2+^ plays an essential role as a ubiquitous and versatile signaling molecule that regulates a wide variety of cellular processes^19^, including muscle contraction, neurotransmission, and hormone secretion. It also acts as an enzyme cofactor and second messenger in organelle communication, cell motility, and cell growth. Because Ca^2+^ exhibits critical and diverse functions, the intracellular Ca^2+^ concentration is tightly regulated^18,20^. The intracellular Ca^2+^ level is maintained at lower levels (50–100 nM) than the extracellular Ca^2+^ level (1–2 mM)^60^. Regulation of intracellular Ca^2+^ levels is controlled by plasma membrane channels, such as nonselective transient receptor potential (TRP) channels, purinergic ionotropic receptors (P2RXs), and voltage-activated Ca^2+^ (Ca_V_) channels, and by ER and mitochondrial Ca^2+^ transporters and pumps, such as plasma membrane Ca^2+^ ATPases (PMCAs), sarcoplasmic/ER Ca^2+^ ATPases (SERCAs), and the mitochondrial Na^+^/Ca^2+^/Li^+^ exchanger (NCLX)^21^ (Fig. 4). PMCAs^61,62^ and SERCAs^63^ pump Ca^2+^ from the cytosol into the extracellular space and the ER, respectively. A decrease in the Ca^2+^ level in the ER lumen is sensed by stromal interaction molecule 1 (STIM1) and STIM2. STIMs activate ORAI and C-type transient receptor (TRPC) proteins at the plasma membrane and induce store-operated Ca^2+^ entry (SOCE)^64^, which increases the rate of intracellular Ca^2+^ entry from the extracellular space. After Ca^2+^ enters the cytosol, SERCAs pump Ca^2+^ into the ER to replenish the Ca^2+^ stores in the ER^65^.

Intracellular Ca^2+^ signaling is initiated by stimuli that increase the level of intracellular Ca^2+^, which then regulates downstream Ca^2+^ signaling pathways. Except for that in neuronal cells, Ca^2+^ signaling in most cells is initially regulated by hormone binding to a G protein-coupled receptor (GPCR). For example, glucagon and catecholamines such as epinephrine activate the stimulatory G protein α (Gαs), leading to stimulation of adenylate cyclase, which produces cAMP^66^. An increase in cAMP level activates protein kinase A (PKA), which further phosphorylates and activates inositol trisphosphate receptors (IP3Rs), resulting in Ca^2+^ release from the ER^67^. Catecholamines such as norepinephrine activate Gαs and further activate PLC, which catalyzes the hydrolysis of PI(4,5)P_2_ to produce the intracellular messengers I(1,4,5)P_3_ and DAG. I(1,4,5)P_3_ binds IP3Rs, stimulating Ca^2+^ release from the ER^68^.

A hormone-stimulated elevation in intracellular Ca^2+^ activates Ca^2+^-dependent signaling molecules such as Ca^2+^ calmodulin (CaM)-dependent kinases (CaMKs)^69^, calcineurin^70^, and Ca^2+^dependent protein kinase C (PKC)^19^ (Fig. 4). CaM undergoes a conformational change following Ca^2+^ binding to its four EF-hand domains, resulting in CaMK activation. Activated CaMKs directly phosphorylate and activate transcription factors, including FOXO, CREB, p38, nuclear factor kappa-light-chain-enhancer of activated B cells (NF-κB), and c-Jun N-terminal kinase (JNK), which are important for metabolism, inflammation, and other cellular processes^69^ (Fig. 4). An elevated intracellular Ca^2+^ level also leads to the activation of calcineurin, a serine/threonine protein phosphatase, which dephosphorylates CREB-regulated transcription coactivator 2 (CRTC2), myocyte enhancer factor-2 (MEF-2), nuclear factor of activated T cells (NFATc), and transcription factor EB (TFEB)^70^. Activated CRTC2, MEF-2, NFATc, and TFEB are translocated from the cytosol to the nucleus, where they activate the expression of target genes essential for metabolic, cardiovascular, immune, lysosome biogenesis, and autophagy functions^21^. When Ca^2+^ and DAG levels are increased in the cytosol, these proteins bind to the C2 and C1 domains of PKC, respectively; PKC is then translocated to the plasma membrane, resulting in its activation. Activated PKC phosphorylates substrates such as mitogen-activated protein (MAP) kinase, transcription factor inhibitor IκB, vitamin D3 receptor (VDR), and calpain^21^.

## Dysregulation of Ca^2+^ channels and pumps in metabolic diseases

**Fig. 5:**
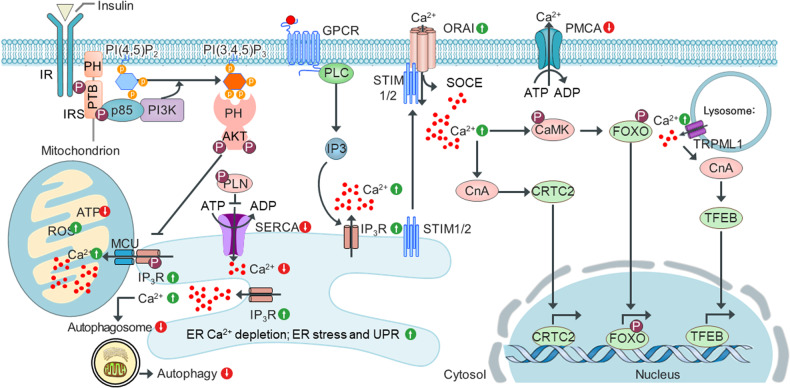
Dysregulation of Ca^2+^ channels and pumps in metabolic diseases. Under normal physiological conditions, insulin-stimulated PI3K phosphorylates PI(4,5)P_2_ to produce PI(3,4,5)P_3_. PI(3,4,5)P_3_ recruits PH domain-carrying AKT to the plasma membrane, where activated AKT phosphorylates IP3Rs, reducing intracellular Ca^2+^ release from the ER. Alternatively, AKT phosphorylates PLN, a critical negative regulator of SERCAs in the ER, thereby promoting SERCA activity and decreasing intracellular Ca^2+^ levels by transporting Ca^2+^ from the cytosol to the ER lumen. Under pathological conditions, such as obesity and hyperlipidemia, however, metabolic stress may increase ORAI and IP3R expression, leading to intracellular Ca^2+^ overload. In the mitochondria, Ca^2+^ overload correlates with increased ROS levels and decreased ATP production. Reduced ATP production may decrease SERCA and PMCA expression and activity, contributing to the depletion of ER Ca^2+^ and increasing intracellular Ca^2+^ and mitochondrial Ca^2+^ overload. In addition, ER Ca^2+^ depletion may increase the UPR and ER stress response. Thus, ER Ca^2+^ depletion and mitochondrial Ca^2+^ overload may exacerbate intracellular Ca^2+^ overload. Increased intracellular Ca^2+^ also decreases autophagosome biogenesis and decreases the autophagy rate. Moreover, increased intracellular Ca^2+^ activates CnA to dephosphorylate CRTC2, promoting its nuclear translocation, and activates CaMK to phosphorylate FOXO, leading to increased gluconeogenesis in the liver. In metabolic diseases, Ca^2+^ is released from lysosomes through TRPML (transient receptor potential cation channel, mucolipin subfamily, member), leading to local CnA activation and TFEB dephosphorylation. Dephosphorylated TFEB transcriptionally activates the lysosomal/autophagic pathway. The resulting ER Ca^2+^ depletion and mitochondrial Ca^2+^ overload may trigger a vicious cycle of intracellular Ca^2+^ overload, which contributes to the development of insulin resistance and other metabolic disorders by activating Ca^2+^-dependent cellular pathways.

Two organelles are important for maintaining intracellular Ca^2+^ levels: the ER and mitochondria. The plasma membrane Ca^2+^ ATPase (PMCA) and Na^+^/Ca^2+^ exchanger (NCX) are the two main regulators of intracellular Ca^2+^; both remove intracellular Ca^2+^ from cells^21^. Thus, intracellular ER and mitochondrial Ca^2+^ homeostasis are orchestrated by transporters and pumps, including PMCAs, SERCAs, and mitochondrial NCLX. Notably, PMCA and SERCA ion pumps require ATP hydrolysis to pump Ca^2+^ ions from the cytosol to the extracellular space and ER (Fig. 5).

Insulin regulates intracellular Ca^2+^ levels via AKT activation, which results in direct phosphorylation of IP3Rs in the ER membrane, inhibiting Ca^2+^ release from the ER, attenuating the decrease in intracellular Ca^2+^ levels, and increasing the ER Ca^2+^ level^71^. AKT-mediated phosphorylation of IP3Rs also protects cells from apoptosis by reducing intracellular Ca^2+^ flux from the ER^72^. A meta-analysis of genome-wide association study (GWAS) data showed that a single-nucleotide polymorphism (SNP) in IP3R2 was associated with alterations in the waist-to-hip ratio adjusted for body mass index (BMI)^72^. IP3R protein levels were increased in a mouse model of obesity (db/db mice), resulting in increased intracellular Ca^2+^ release from the ER to the cytosol^73^ and CaMK activation^26^ (Fig. 5). Furthermore, IP3R1 inhibition in the liver enhanced glucose metabolism in obese mice^73^, highlighting the significance of IP3Rs in human health and metabolic disease.

AKT directly interacts with phospholamban (PLN)^74^, a critical negative regulator of SERCAs in the ER^8^, and phosphorylates PLN at Thr17, promoting SERCA activity and resulting in increased Ca^2+^ reuptake from the cytosol to the ER. Thus, phosphorylation of PLN by AKT stimulates SERCA activity, decreasing the intracellular Ca^2+^ level. Moreover, alterations in the ER fatty acid and lipid compositions in the obesity context inhibited SERCA activity and ER stress, leading to Ca^2+^ depletion in the ER and impaired protein folding capacity^24^ (Fig. 5). These alterations have important implications for inflammatory signaling and the development of insulin resistance. SERCA overexpression increased the ER folding capacity and re-established glucose homeostasis in ob/ob mice^24^, highlighting the importance of SERCAs in the development of fatty liver disease and diabetes.

PMCA is an ATP-driven Ca^2+^ pump that is critical for maintaining a low intracellular Ca^2+^ level in resting eukaryotic cells. Impaired PMCA activity and reduced PMCA expression lead to intracellular Ca^2+^ overload and Ca^2+^-dependent cell death^75^. PMCA activity is inhibited by ROS and ATP depletion under metabolic stress conditions^76^. In contrast, PI(4,5)P_2_, a major PMCA activator, increases the affinity of PMCA for ATP and that of Ca^2+^/CaM for an autoinhibitory motif; PMCA then increases Ca^2+^ movement into the extracellular space^76^. Importantly, a role of the PMCA pump in human metabolic disease has been suggested by a recent GWAS-related meta-analysis that revealed an SNP in the PMCA1 locus associated with alterations in type 2 diabetes and HbA1c-adjusted BMI (Fig. 5). Reduced PMCA1 expression is associated with increased blood pressure, elevated cytosolic Ca^2+^ levels, and vascular remodeling^77^. PMCA2 deficiency is associated with hearing loss/deafness or ataxia in humans and mice^62^. Moreover, a GWAS data-based meta-analysis revealed SNPs in multiple PMCA3 loci associated with systolic and diastolic blood pressure and hypertension^62^. Loss of PMCA4 leads to Ca^2+^ overload and apoptosis under some conditions^62^, highlighting the importance of PMCA Ca^2+^ pumps in human health and disease.

Disruptions to Ca^2+^ homeostasis in the ER, mitochondria, lysosomes, and endosomes play key roles in obesity and insulin resistance^18,20^. Levels of free fatty acids (FFAs), especially saturated palmitic acid (PA), are significantly higher in obese individuals than in healthy subjects^78^. Increased serum FFA levels are closely linked to obesity-associated metabolic complications^79,80^, such as insulin resistance, type 2 diabetes, and nonalcoholic fatty liver disease (NAFLD). Numerous studies^81–85^ have shown that high PA levels induced Ca^2+^-mediated pathogenic changes, such as ER stress and lysosomal and mitochondrial dysfunction. High PA levels trigger aberrant ER Ca^2+^ release, thereby depleting ER Ca^2+^ stores. ER Ca^2+^ deficiency impairs the protein folding machinery, which leads to the UPR and accumulation of misfolded proteins. PA-induced ER Ca^2+^ deficiency leads to mitochondrial Ca^2+^ overload, reduces the mitochondrial membrane potential, and increases ROS levels in various cell types^27,81–84^ (Fig. 5). Importantly, treatment with the Ca^2+^ chelator BAPTA has been reported to attenuate PA-induced lipotoxicity in hepatocytes^22^. Moreover, SOCE triggered by the loss of ER Ca^2+^ further exacerbates persistent intracellular Ca^2+^ overload^27^. Thus, obesity- or PA-induced dysregulation of ER and/or mitochondrial function results in a vicious cycle that aggravates mitochondrial and intracellular Ca^2+^ overload, leading to abnormal insulin action and metabolic alterations^27^. PA-induced elevation of intracellular Ca^2+^ has been shown to attenuate autophagic flux by inhibiting autophagosome and lysosome fusion^86^ (Fig. 5). Moreover, treatment with the Ca^2+^ channel blocker verapamil attenuated ER stress and reduced autophagic flux and glucose metabolism in obese animals^86,87^. These findings suggest that intracellular Ca^2+^ overload is a critical link among ER, mitochondrial stress, and autophagy in the context of obesity.

Lysosomal Ca^2+^ homeostasis dysregulation can interfere with autophagy, damage mitochondrial functionality, and trigger inflammation^88^. This disruption also leads to altered lipid metabolism and impacts endocytosis and exocytosis^89^. These changes contribute to the development and progression of metabolic disorders such as obesity, type 2 diabetes, and nonalcoholic fatty liver disease^88–90^. Moreover, the regulation of the transient receptor potential cation channel, mucolipin subfamily, member 1 (TRPML1) by various phosphoinositides, a group of signaling lipids, is of particular interest. TRPML1 activity is known to be positively modulated by PI(3,5)P_2_, while other PIPs, such as PI(3,4)P_2_, PI(4,5)P_2_, and PI(3,4,5)P_3,_ inhibit its function, affecting the release of Ca^2+^ from lysosomes into the cytosol^91^. Under conditions such as obesity, in which the intracellular Ca^2+^ level is elevated, PIPs such as PI(3,4)P_2_, PI(4,5)P_2_, and PI(3,4,5)P_3_ form Ca^2+^-PIPs^29^, which potentially counteract the inhibitory effects on TRPML1, leading to sustained release of lysosomal Ca^2+^ into the cytosol. Disruption of TRPML1 regulation by these phosphoinositides significantly inhibits cellular functions and may lead to various diseases.

Given these findings, the obesity-associated dysregulation of intracellular Ca^2+^ homeostasis is a key mechanism in metabolic tissues, which, in turn, leads to organelle dysfunction. In the context of obesity, the disruption of Ca^2+^ homeostasis in the ER and mitochondria leads to ER stress, impaired protein folding, and reduced mitochondrial functioning, including reduced mitochondrial ATP production and increased mitochondrial ROS production^28^. The resulting ER and mitochondrial dysfunction may trigger a vicious cycle of intracellular Ca^2+^ homeostasis disruption, causing the downregulation of ATP-driven Ca^2+^ pumps such as SERCAs and PMCAs and thus leading to intracellular Ca^2+^ overload (Fig. 5).

## Ca^2+^-mediated inhibition of membrane localization of PIP-binding proteins

Dysregulation of Ca^2+^ homeostasis is associated with metabolic diseases such as obesity, insulin resistance, and type 2 diabetes. Intracellular Ca^2+^ overload has been reported in primary adipocytes isolated from diabetic rats^92^ and obese patients with insulin resistance^93^. Lipid-induced chronic alterations in Ca^2+^ concentrations in the cytosol, ER, and mitochondria have been observed in the liver, muscle, and adipose tissue of dietary and genetic mouse models of obesity, obese humans, and those with NAFLD^18–20,28^. Moreover, intracellular Ca^2+^ overload increases ER stress, the UPR, mitochondrial ROS levels, and oxidative stress^94^, leading to impaired carbohydrate, lipid, and protein metabolism^24,25,28^. Intervention with the Ca^2+^ channel blocker verapamil increased insulin sensitivity and glucose homeostasis in obese model animal^86,87^. A human clinical trial revealed that verapamil reduces external insulin requirements and results in improved blood sugar control in adults with recent-onset type 1 diabetes^95^. Recent studies have demonstrated that BAPTA-AM, a cell-permeable Ca^2+^ chelator, shows promising therapeutic effects on acute kidney^96^ and liver failure^4^ because it abolishes excessive intracellular Ca^2+^ levels. These results suggest that restoring intracellular Ca^2+^ to normal levels may be a promising therapeutic strategy in the management of insulin resistance and metabolic disease. However, the molecular mechanisms that link intracellular Ca^2+^ overload to insulin resistance and metabolic disease have not yet been fully elucidated.

**Fig. 6:**
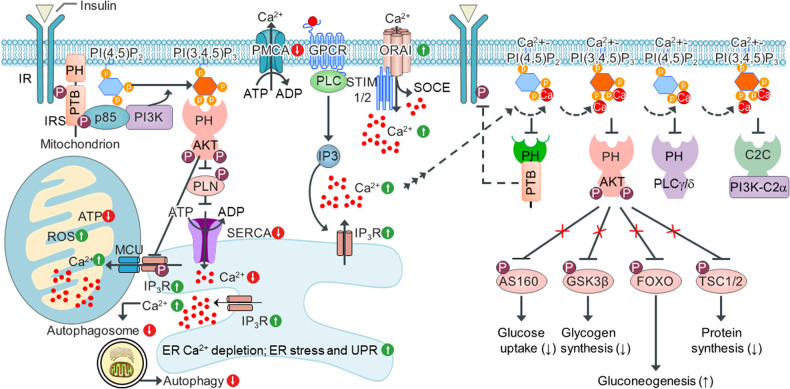
Ca^2+^-mediated inhibition of membrane localization of PIP-binding proteins. Under pathological conditions such as obesity and hyperlipidemia, metabolic stress can increase the expression of Ca^2+^ channel proteins such as ORAI and IP3R, thereby increasing the intracellular degree of Ca^2+^ overload. At the same time, the expression and activity of SERCAs and PMCAs may decrease, causing depletion of Ca^2+^ stores in the ER and increased intracellular and mitochondrial Ca^2+^ overload. In addition, dysregulation of intracellular Ca^2+^ homeostasis results in ER stress, activation of the UPR, increased ROS production, and oxidative stress, all of which may further impair the function of ATP-driven Ca^2+^ pumps such as SERCAs and PMCAs. The resulting ER and mitochondrial dysfunction can trigger a vicious cycle of intracellular Ca^2+^ overload and contribute to the development of insulin resistance and other metabolic disorders. In addition to affecting several Ca^2+^-dependent cellular pathways, such as the CaMK and CaN pathways, increased intracellular Ca^2+^ levels cause Ca^2+^ to bind PIPs and thus form Ca^2+^-PIPs, which disrupt the electrostatic interactions between PIPs and PH domains or C2C domains in proteins, inhibiting the membrane localization of PH domain- or C2C domain-carrying proteins, and disrupting PIP signaling. Thus, Ca^2+^-PIP mediates the inhibition of the membrane localization of PH domain- or C2C domain-carrying signaling proteins, which leads to modulated signaling pathway activation in response to changes in the intracellular Ca^2+^ level.

We recently identified a novel physiological role for intracellular Ca^2+^ functioning as a critical negative regulator to prevent the interaction between the PH domains in AKT, PLCδ, IRS1 with PIPs during insulin signaling^29^. Ca^2+^ directly binds two adjacent phosphorylated head groups in PIPs, including PI(3,4)P_2_, PI(4,5)P_2_, and PI(3,4,5)P_3_, at physiological concentrations. This binding results in the formation of Ca^2+^-PIPs such as Ca^2+^-PI(3,4)P_2_, Ca^2+^-PI(4,5)P_2_, and Ca^2+^-PI(3,4,5)P_3_, which inhibit the electrostatic interaction between PH domains and membrane PIPs, ultimately preventing membrane targeting of PH domain-carrying proteins and disrupting insulin signaling^29^ (Fig. 6). These results are consistent with the known characteristics of inositol phosphates, which form a bidentate structure with a Ca^2+^ and two adjacent phosphate groups in inositol phosphates^97^. Furthermore, molecular dynamic simulations have revealed an interaction of intracellular Ca^2+^ ions with phosphorylated head groups of PI(4.5)P_2_^98^; this interaction inhibits membrane localization of PH domain-carrying PLCδ to a membrane PI(4,5)P_2_. In addition to the PH domain-carrying proteins, the Ca^2+^-independent C2C domain in PI3K-C2α is recruited to the plasma membrane by PI(4,5)P_2_ and PI(3,4,5)P_3_ under normal conditions. In contrast, higher intracellular Ca^2+^ levels prevent the membrane localization of Ca^2+^-independent C2C domain-carrying PI3K-C2α by forming Ca^2+^-PI(4,5)P_2_ and Ca^2+^-PI(3,4,5)P_3_^30^. Additional compelling experiments confirmed that a high intracellular Ca^2+^ level inhibits membrane localization of the PH domain-carrying AKT and PLCδ^30^. These data suggest that a high intracellular Ca^2+^ level is important for inhibiting the membrane localization of PH domain-carrying AKT, PLCδ, and IRS1, as well as Ca^2+^-independent C2C domain-carrying PI3K-C2α, indicating a novel role in regulating Ca^2+^ and PIP-mediated signaling. Because both Ca^2+^ and PIPs are versatile and specific signaling factors associated with many regulatory pathways in a cell, the formation of Ca^2+^-PIPs when the concentration of intracellular Ca^2+^ is high also suggests that Ca^2+^ prevents membrane localization of PIP-binding proteins such as the PH domain–carrying proteins AKT, PLCδ, and IRS1 and the Ca^2+^-independent C2C domain–carrying protein PI3K-C2α because Ca^2+^ is coupled to PIPs (Fig. 6). Finally, we highlighted the multifaceted role of intracellular Ca^2+^ overload in the development of insulin resistance in both obese animals and PA-treated HepG2 cells^99^. These studies provided compelling evidence that the antihypertensive agent candesartan, a drug that inhibits obesity-associated intracellular Ca^2+^ overload and lipid accumulation, ameliorated obesity-induced insulin resistance by enhancing the postprandial membrane localization of PH domain-carrying AKT, leading to increased insulin sensitivity and signaling.

## Conclusions and perspectives

This review provides an overview of Ca^2+^ homeostasis and PIP-binding signaling proteins, which are versatile signaling molecules associated with many regulatory cellular pathways. PIPs constitute a family of seven interconvertible phospholipids found in mammalian cells. PIPs play critical roles in recruiting PH domain–carrying proteins to the plasma membrane, thereby regulating diverse cellular functions, including cell growth and survival, vesicular trafficking, and cytoskeleton reorganization. The PI3K/AKT signaling pathway is an important intracellular signaling pathway that is activated by various stimuli, including growth factors, cytokines, and cellular stress. Upon activation, the PI3K enzyme phosphorylates PI(4,5)P2 to generate PI(3,4,5)P3. PI(3,4,5)P3 then recruits PH domain–carrying signaling proteins, such as PDK, AKT, GTPases (such as Rac and Rho), ITK, and BTK, to the plasma membrane. These PH domain–carrying proteins are then activated at the plasma membrane and regulate a variety of cellular functions, including cell survival, proliferation, cytoskeleton rearrangement, intracellular vesicle trafficking, and cell metabolism.

Ca^2+^ homeostasis is maintained by the actions of transporters and pumps, including PMCAs, SERCAs, and NCLX. These proteins ensure that the proper Ca^2+^ levels are maintained in the ER and mitochondria, organelles that play critical roles in cellular metabolism and signaling. SERCAs are located in the ER and pump Ca^2+^ ions into the ER compartment, while PMCAs are located in the plasma membrane and pump Ca^2+^ out of the cell. Moreover, studies have shown that dysregulation of SERCAs and PMCAs results in increased intracellular Ca^2+^ levels and alterations in Ca^2+^ homeostasis in various metabolic diseases, including diabetes, cardiovascular disease, and obesity. These findings highlight the importance of understanding the mechanisms involved in regulating intracellular Ca^2+^ levels and their effects on cellular signaling. Several compelling pieces of evidence presented herein demonstrate that our comprehension of PIPs and Ca^2+^ coupling is particularly important given the consequences of intracellular Ca^2+^ dysregulation in diverse pathological settings, including cardiovascular disease, insulin resistance, and type 2 diabetes. One important discovery in the field of PIPs and intracellular Ca^2+^ signaling is the role of Ca^2+^-mediated inhibition of the membrane localization of PIP-binding proteins such as those carrying PH domains and C2C domains, suggesting a novel mechanism by which PIP-binding proteins are regulated on the basis of the intracellular Ca^2+^ level. This mechanism has been shown to regulate various cellular functions, including endocytosis, intracellular trafficking, and gene expression. These findings provide new insights into the complex interplay between PIPs and intracellular Ca^2+^ signaling and highlight the importance of understanding these relationships for developing new therapeutic strategies for treating metabolic diseases. Because the regulation of intracellular Ca^2+^ and PIP signaling is a complex and dynamic process that is critical to maintaining cell signaling, disruption to these signaling processes can have unintended consequences. Therefore, it is important to carefully consider the potential risks and benefits before developing therapeutic strategies targeting these pathways in patients with obesity, diabetes, or other chronic metabolic diseases.
